# The Immunology of Zoonotic Infections

**DOI:** 10.1155/2012/208508

**Published:** 2012-02-26

**Authors:** Georgios Pappas, Antonio Cascio, Alfonso J. Rodriguez-Morales

**Affiliations:** ^1^Institute of Continuing Medical Education of Ioannina, 45333 Ioannina, Greece; ^2^Working Group on Zoonoses, International Society of Chemotherapy, UK; ^3^Tropical and Parasitological Diseases Unit, Department of Human Pathology, University of Messina, 98122, Messina, Italy; ^4^Department of Preventive and Social Medicine, Luis Razetti Medical School, Faculty of Medicine, Universidad Central de Venezuela, Caracas 1050, Venezuela; ^5^Infection and Immunity Research Group, Faculty of Health Sciences, Universidad Tecnológica de Pereira, 660001 Pereira, Risaralda, Colombia

Zoonotic infections are in general defined as infections transmitted from animal to man (and less frequently vice versa), either directly (through contact or contact with animal products) or indirectly (through an intermediate vector as an arthropod or an insect) [1]. Although the burden of zoonotic infections worldwide is major, both in terms of immediate and long-term morbidity and mortality [2, 3] and in terms of emergence/reemergence and socioeconomical, ecological, and political correlations [4], scientific and public health interest and funding for these diseases remain relatively minor. 

Zoonoses include diseases induced by diverse pathogens (bacteria, viruses, fungi, and parasites), but a common pattern for the majority of them is their complexity: this term refers not only to their ecology, range of clinical characteristics, and diagnostic and therapeutic challenges, but foremost to their immunology. In fact, all other ecological, clinical, diagnostic and therapeutic complexities emerge from this multifaceted zoonotic pathophysiology, as certain papers of this special issue outline. 

The paper by A. Agnone et al. in the present special issue underlines the complexity, as well as the derived therapeutic and diagnostic significance, of antigen-specific T-cell immune response in varying zoonotic infections. The authors underline the importance and difficulty of understanding these complex pathogenetic mechanisms, as well as their significance for the development of preventive vaccines. The paper by P. Skendros and I. Mitroulis focuses on another specific and increasingly recognized as important part of zoonotic pathophysiology, that of autophagic response in certain intracellular zoonoses, outlining how this autophagic machinery can be exploited by zoonotic pathogens, typically culminating in chronic infections. 

Our ability to understand pathogenetic mechanisms in the subcellular level has greatly evolved in recent years, and the paper by P. Mansueto et al. demonstrates how this progress has allowed us to extensively understand the intracellular interactions observed in rickettsial infections, a group of zoonoses that includes diverse pathogens with certain common characteristics. The paper by C. F. Oliveira et al. is one of the papers in this special issue attempting to translate theoretical knowledge in experimental data: the authors demonstrate in a mice model how a particular cytokine receptor deficiency induces a specific default in immunity against *Leishmania major* that results in specific clinical manifestations. 

Other three papers all deal with the immunology of cystic echinococcosis (CE), a worldwide prevalent parasitic zoonosis with major public health burden: the paper by W. Zhang et al. summarizes all novel developments in the understanding of host immune responses in CE and proposes potential translation of this understanding in the diagnostic field. The paper by A. Siracusano et al. adds a different angle at our understanding of these mechanisms and discusses how the evolution of proteomics would further enhance our pathogenesis understanding. The paper by L. Piccoli et al. focuses on an aspect of immunologic diagnosis and activity evaluation of CE, outlining the difficulties such approaches may impose. 

There are three papers that focus on parameters of the immunology of brucellosis, possibly the dominant bacterial zoonotic infection worldwide and known to induce a potentially noneradicable disease. The paper by F. S. Oliveira et al. is the first to demonstrate the critical role and the specific nature of nucleotide-binding oligomerization domain (NOD) receptors in the immune response against *Brucella abortus*. The paper by E. D. Avila-Calderón et al. focuses on the potential utility of certain outer membrane vesicles of *B. melitensis *in vaccine development, using an experimental mice model, while the paper by G. C. Macedo et al., in a similar experimental model, evaluates the exact role of interferon gamma in host protection against *B. abortus*. 

Other two papers focus on viral zoonoses: the paper by J. Juno et al. is an up-to-date review of our knowledge of both intrinsic and pathogen-related immunomodulating factors affecting susceptibility to and clinical severity of pandemic H1N1 and avian influenza (H5N1) infection, while the paper by J. A. Silva Gomes et al. discusses the peculiarities of immune response induced by vaccinia virus (an orthopoxvirus similar to smallpox) both in humans naturally infected and in an experimental model. 

The paper by M. Mosepele et al. focuses on the peculiarities of *Bartonella* infections outlining our limited knowledge of specific pathogenetic events taking place in immunocompromised patients, a subgroup of patients that is growing and is growingly susceptible to both *B. quintana* and *B. henselae*. 

Altogether, these papers underline the multifactorial nature and the multiple potential applications of zoonotic pathophysiology knowledge. They further underline the need for enhanced scientific interest (also translated in enhanced funding) in zoonotic pathophysiology: this interest would not only allow the expansion of our theoretical knowledge but would eventually allow for improved understanding of host susceptibility, and thus prevention, improved diagnosis (since zoonoses do not fall into the typical microbiological diagnostic terms of culture positivity or eradication), and possibly novel therapeutic or preventive approaches through development of sophisticated vaccines. 

**Georgios Pappas** **Antonio Cascio** **Alfonso J. Rodriguez-Morales**
